# Rethinking diabetes in the United States

**DOI:** 10.3389/fendo.2023.1185719

**Published:** 2023-06-15

**Authors:** William H. Herman, Dean Schillinger

**Affiliations:** ^1^ Department of Internal Medicine, Division of Metabolism, Endocrinology, and Diabetes, University of Michigan, Ann Arbor, MI, United States; ^2^ Department of Epidemiology, School of Public Health, University of Michigan, Ann Arbor, MI, United States; ^3^ San Francisco General Hospital, University of California San Francisco School of Medicine, San Francisco, CA, United States

**Keywords:** socioecological model, social determinants of health, diabetes prevention, federal government, health policy

## Abstract

Despite the availability of effective medical treatments, the diabetes epidemic has accelerated in the United States, efforts to translate treatments into routine clinical practice have stalled, and health inequities have persisted. The National Clinical Care Commission (NCCC) was established by the Congress to make recommendations to better leverage federal policies and programs to more effectively prevent and control diabetes and its complications. The NCCC developed a guiding framework that incorporated elements of the Socioecological and Chronic Care Models. It gathered information from both health-related and non-health-related federal agencies, held 12 public meetings, solicited public comments, met with interested parties and key informants, and performed comprehensive literature reviews. The final report of the NCCC was transmitted to the Congress in January 2022. It called for a rethinking of the problem of diabetes in the United States, including the recognition that the lack of progress is due to a failure to confront diabetes as both a complex societal problem as well as a biomedical problem. To prevent and control diabetes, public policies and programs must be aligned to address both social and environmental determinants of health and health care delivery as they impact diabetes. In this article, we discuss the findings and recommendations of the NCCC as they relate to the social and environmental factors that influence the risk of type 2 diabetes and argue that the prevention and control of type 2 diabetes in the U.S. must begin with concrete population-level interventions to address social and environmental determinants of health.

## Introduction

Despite evidence that lifestyle and medication interventions can delay or prevent the development of type 2 diabetes; that safe and effective treatments can control glucose, blood pressure, and lipid levels; and that targeted and appropriately timed interventions can slow the progression and reduce the impact of microvascular, neuropathic, and cardiovascular complications, the diabetes epidemic has accelerated in the United States, efforts to translate treatments into routine clinical practice have stalled, and health inequities have persisted. Between 2003 and 2020, the age-adjusted prevalence of diabetes among U.S. adults 18 years of age and older increased from 10.2% to 13.2% (1). Between 2007 and 2018, the percentage of U.S. adults with diagnosed diabetes achieving treatment goals of glycated hemoglobin <7%, blood pressure <140/90 mmHg, and non-HDL-cholesterol <130 mg/dl decreased from 24.9% to 22.2% (2). And while the age-adjusted incidence of non-traumatic lower extremity amputations in adults with diabetes decreased steadily from 2000 to 2009, the incidence increased progressively after 2010 (3). Recognition of these gaps between what is and what could be led the National Clinical Care Commission (NCCC) to rethink population-wide approaches to the prevention and control of diabetes in the United States.

## Methods

In 2017, the United States Congress passed Public Law 115-80 which established the NCCC. The NCCC was charged with evaluating and making recommendations to the Secretary of Health and Human Services and to Congress regarding improving the coordination and leveraging of programs within the Department of Health and Human Services and other federal agencies related to awareness, prevention, and clinical care for diabetes (4). The NCCC included 23 members. Twelve non-federal members represented physician specialists, primary care physicians, health care providers serving Medicaid and uninsured populations, non-physician health care professionals, patient advocates, and public health experts. Eleven additional members each represented a different federal agency. The NCCC systematically collected information on federal policies and programs relevant to diabetes from both health-related and non-health-related federal agencies. It adopted a framework for its deliberations that combined elements of both the Socioecological Model (5) and the Chronic Care Model (6) and recognized that lack of progress in the prevention and control of diabetes is due to a failure to recognize diabetes as both a societal problem and a complex medical problem. It concluded that to prevent and control diabetes, public policies and programs must be aligned to address both social determinants of health and health care delivery as they impact diabetes (4). The authors of this article were as appointed non-federal members of the NCCC.

To develop evidence-based and actionable recommendations, the NCCC formed three subcommittees focused on 1) population-wide strategies to prevent and control diabetes; 2) targeted diabetes prevention strategies for individuals at risk for type 2 diabetes, including those with prediabetes; and 3) the treatment of diabetes and its complications in individuals with diabetes. In this article, we discuss the findings and recommendations of the NCCC’s General Populations Sub-Committee as they relate to the social and environmental factors that influence both the risk of type 2 diabetes and the management of type 1 and type 2 diabetes. We argue that population-wide interventions to address social and environmental determinants of health are fundamental to diabetes prevention and control in the U.S (7, 8). Other papers have described the recommendations of the two other sub-committees (9, 10).

## Rationale

Americans who have less education, lower incomes, less wealth, food and housing insecurity, and who live in rural areas have higher rates of type 2 diabetes. Rates of type 2 diabetes are higher in neighborhood environments that lack playgrounds, parks, and walkability and in areas where people are exposed to environmental toxins. Poor social cohesion, marginalization, historical trauma, and structural racism also contribute to the diabetes epidemic by increasing exposure to unhealthy environments and conditions (11).

Lower income individuals and racial and ethnic groups that experience higher diabetes prevalence also have higher rates of preventable, severe, and costly complications. Social and environmental factors are associated with self-management of diabetes and improvements in diabetes outcomes have not been evenly distributed across the United States population (12). Poor glycemic control and poor blood pressure control are both more frequent among poor and uninsured people with diabetes than among wealthier and insured people with diabetes (13). Compared to adults with higher incomes, U.S. adults with lower incomes report skipping 23% more doctor visits, tests, treatments, or prescription medications because of cost (14). Non-adherence to medical care due to cost has been reported in 20% to 40% of people with diabetes. For those with self-reported financial insecurity, the non-adherence rate can be as high as 60% (15).

Recently, there has been increasing recognition that social and environmental factors influence health. A recent White House conference on Hunger, Nutrition and Health endorsed medically tailored meals as a first step in addressing food insecurity and nutritional quality as social determinants of health (16). The “food as medicine” concept had its roots in the AIDS epidemic when volunteers delivered meals to patients to prevent the cachexia that individuals with AIDS experienced. In the past few years, the concept of “food as medicine” has been popularized by Medicare Advantage plans that include some variation of home-delivered meals to meet the needs of patients with conditions as diverse as diabetes, kidney disease, and heart failure. A number of states have also offered medically tailored meals through Medicaid under wavers from the Department of Health and Human Services. In one study of over 1,000 adults, weekly delivery of ten ready-to-eat meals tailored to the specific medical needs of the individual under the supervision of a registered dietitian was associated with significantly fewer inpatient admissions, fewer skilled nursing facility admissions, and lower costs (17).

Although medically tailored meals provided by health systems can address food insecurity as a contributor to diabetes and its complications, they are limited in their scope and tend to “medicalize” what is in fact a social issue. Non-health-related federal departments and agencies are responsible for policies and programs that impact food, education, housing, transportation, trade, commerce and the environment, and have an enormous role in shaping social and environmental conditions that influence population health. The NCCC recognized that implementing changes in the policies and programs of these non-health-related federal agencies and ensuring their cooperation and collaboration with federal agencies that are accountable for health care concerns offers the greatest promise in addressing diabetes in the United States.

While some countries have affirmatively addressed diabetes through trans-sectoral governmental activities, the U.S. has not (18). The U.S. generally lacks structures to coordinate strategic planning across non-health-related and health-related federal agencies. Indeed, many non-health-related federal agencies may inadvertently implement policies and programs that are antithetical to the missions and objectives of health-related federal agencies. A health-in-all policies (HiAPs) approach can address the complex factors that influence health and equity by articulating and integrating health considerations into policy-making across diverse sectors (19). A HiAPs approach takes into account the health implications of policy decisions, seeks synergies between non-health-related and health-related agencies, and avoids harmful health impacts and health inequities that can unintentionally arise from the policies and practices of non-health-related agencies.

Health impact assessments (HIAs) are tools by which policies and programs may be judged as to their potential effects on the health of populations and the distribution of health across populations (18). HIAs are an evidence-based method to promote the HiAP approach. The NCCC recognized that sustained national efforts to adopt a HiAP approach and to mandate HIAs could do much to address social determinants of health and facilitate the prevention and control of diabetes in the United States. Indeed, the federal government can play a larger role in preventing and controlling diabetes by ensuring that non-health-related federal agencies conduct HIAs and consider their results when implementing policies and programs. A number of examples, taken from the Report of the National Clinical Care Commission, follow (4). In the remainder of this report, we describe recommendations made by the NCCC General Populations Sub-Committee, whose purpose was to identify agency actions that affect health risk in the overall population including those without diabetes, those at risk for diabetes, and those with diabetes (4).

## Recommendations

### Nutrition, food policy, and clean drinking water

Many policies and programs of the United States Department of Agriculture (USDA) profoundly affect the nutritional status of Americans. In fiscal year 2021, the Special Supplemental Nutrition Program for Women, Infants, and Children (WIC) served approximately 6.2 million low-income women, infants, and children each month (20, 21). WIC seeks to ensure that low income pregnant and postpartum women and their children up to age 5 have access to nutritious foods, that women deliver infants with appropriate birth weights, that women receive breastfeeding support, and that their children achieve appropriate BMI percentiles. Unfortunately, enrolling in WIC can be difficult, and the proportion of eligible people who participate in WIC is only 57% (22). Recent improvements to the nutritional content of the WIC package have been shown to be beneficial to population health (23). By updating the technology infrastructure of WIC and increasing participation among eligible women, participants could be enabled to buy and consume more fruits and vegetables and breastfeeding could be promoted as the optimal infant feeding choice (7, 8).

The USDA Supplemental Nutritional Assistance Program (SNAP) provides ~$80 billion per year to address food insecurity and improve access to foods and beverages for approximately 42 million lower-income Americans each year (24). SNAP is a valuable program for reducing food insecurity, but its impacts on diet quality and diabetes risk have not been optimized. For example, in 2016, SNAP households spent approximately $4 billion of SNAP resources to purchase sugar-sweetened beverages (SSBs) (25). By expanding outreach to enable all SNAP-eligible individuals to receive SNAP benefits, increasing the benefit to better reflect the current prices of healthy foods in today’s marketplace, providing incentives for the purchase of fruits and vegetables, removing SSBs as an allowable SNAP purchase, and expanding educational efforts to reduce nutrition-related diabetes risks, the USDA could better address the nutritional needs of Americans and contribute to the prevention and control of diabetes (7, 8).

Sugar-sweetened beverages (SSBs) represent the largest single source of added sugar in the American diet (30-40%) and account for 50-90% of the recommended daily limit of added sugars (26). The highest intake of SSBs occurs among adolescents, non-Hispanic Black and Hispanic people, and groups with lower socioeconomic status (27). To address this issue, the USDA should require schools to ban the sale of SSBs in order to receive funding through the National School Lunch and Breakfast Program (see below). At the same time, the Departments of Education and the Environmental Protection Agency (EPA) could collaborate to ensure that free and clean water is accessible on all school campuses. Congress could also enact an excise tax of as little as 1 cent per ounce (about 10% of the price) on the cost of SSBs to reduce consumption. The revenues from the tax could then be used to fund health promotion activities including access to safe drinking water (7, 8). Currently, six U.S. localities levy taxes on SSBs (Albany, Berkley, Oakland, and San Francisco, CA, Boulder, CO, and Seattle, WA). Philadelphia, PA also levies a per-volume soda excise tax on purchases of soft drinks. Evaluation of these taxes and similar taxes imposed in the United Kingdom (U.K.) and Mexico have demonstrated that they are associated with reduced SSB consumption (28–31). A recent evaluation of the health impact of the U.K. tax has shown reductions in obesity among children (32).

The USDA also supports the National School Lunch and Breakfast Program which together serve approximately 30 million children each day (33). The Summer Food Services Program and Seamless Summer Option, federally-funded, state-administered programs that reimburse not-for-profit community organizations to serve free, healthy meals to children and teens in low-income communities during the summer are also supported by the USDA (34). Providing these programs sufficient financial resources to offer foods that meet nutritional standards and expanding the summer meal programs to serve more of the low-income children served by the National School Lunch and Breakfast Program could help to address childhood obesity and prevent type 2 diabetes in youth, which is a growing clinical and public health problem 7,8].

Recently, the USDA added $40 million to a Farm Bill Program called the Gus Schumacher Nutrition Incentive Program (GusNIP), the fresh fruit and vegetables program, to scale up the use of “produce prescriptions” (PRx). PRx are similar to medically tailored meals but provide fresh produce to at-risk individuals either in boxes or through vouchers or debit-type cards. Unlike medically tailored meals, the health impact of PRx has not been rigorously evaluated, but they represent an important first step in marshalling what are traditionally viewed as “non-health-related federal agencies” to improve population health (35). The USDA Farm Bill ($86 billion per year) (36) can be further harnessed to better prevent and control diabetes and reduce disparities by increasing funding to three programs: the Specialty Crop Block Grant Program that targets the cultivation of fruits, vegetables, and tree nuts; the Specialty Crop Research Initiative that addresses the sustainability of the specialty crop industry; and the Healthy Food Financing Initiative that provides grants and loans to improve access to fresh and healthy foods in low-income settings (7, 8).

### Food and beverage labeling and marketing

The Food and Drug Administration (FDA) can also be enlisted to improve the nutritional status of the general population by improving food and beverage labeling and limiting misleading product claims. The general public, especially individuals with lower education and income levels, are frequently misinformed about the nutritional value and health risks of foods and beverages (37). Inaccurate and misleading marketing claims about health benefits (such as “whole grain”, “low fat”, and “real”) make it difficult for individuals to accurately identify health risks and make informed food choices. By requiring clear, direct, and compelling food and beverage labeling – such as traffic light icons –to inform consumers’ dietary choices, the FDA can contribute to improving the nutritional status of Americans (38). Such an approach has been implemented and proven to be effective in a number of Latin American countries (39, 40). The Federal Trade Commission, if provided the appropriate authority, can also be used to reduce diabetes risk by restricting commercial advertising and marketing of unhealthy foods and beverages to children under the age of 13 years who are unable to objectively evaluate marketing claims (7, 8, 41).

### Promoting breastfeeding

Breastfeeding has been shown to be associated with reduced risk of diabetes among mothers and lower rates of obesity among their offspring (42). Having paid maternity leave for at least 3 months is associated with higher rates, longer duration, and greater intensity of breastfeeding (43). Breast feeding and diabetes prevention can be facilitated by the Department of Labor ensuring that all work sites offer lactation support for breastfeeding mothers. Congress could also enact universal, paid maternity leave for at least 3 months to facilitate persistent breastfeeding (7, 8).

### The built and ambient environments

Attributes of the built and ambient environments also influence diabetes risk and management and are directly subject to government policies and programs (44–47). Housing quality and area-level attributes such as walkability, green spaces, physical activity resources, and opportunities for active transport are determinants of type 2 diabetes risk (48, 49). The Department of Housing and Urban Development (HUD) and the Internal Revenue Service (IRS), through its Low-income Housing Tax Credit Program, can impact the availability and quality of housing for low-income individuals and families and can expand housing opportunities in low-poverty neighborhoods. The landmark Moving to Opportunity Study demonstrated that moving from a neighborhood with a high level of poverty to one with a lower level of poverty was associated with reductions in the incidence of both extreme obesity and diabetes (50). Similarly, the Department of Transportation can implement policies to enhance green spaces, walkability, and opportunities for active transport. The EPA can also ensure that its policies, practices, regulations, and funding decisions lead to environmental changes to prevent and control exposures to air pollution, contaminated water, and endocrine disrupting chemicals that affect diabetes risk (7, 8, 47, 51, 52).

### Coordinating the policies and programs of non-health-related and health-related federal agencies

Finally, there is a need to coordinate and monitor federal efforts to prevent and control diabetes and to ensure trans-agency collaboration among non-health-related and health-related federal agencies. Coordinating the activities of large, non-health-related federal agencies as diverse as USDA, FDA, FTC, the Department of Labor, HUD, IRS, DOT, and EPA with those of health-related federal agencies will be an enormous challenge. An Office of National Diabetes Policy, analogous to the Office of National AIDS Policy, should be created and given responsibility to develop and implement a national diabetes strategy (53, 54).

## Conclusions

While much remains to be done to address diabetes as the complex medical problem that it is, a new kind of work must begin to address the social and environmental factors that influence the risk of type 2 diabetes and impact the management of type 1 and type 2 diabetes. As we have indicated in this report, non-health-related federal departments and agencies are responsible for policies and programs that impact food and agriculture, education, housing, transportation, trade, commerce, and the environment. They play an enormous role in shaping the social and environmental conditions that influence population health. The challenge before us is to implement changes in the policies and programs of these so-called “non-health-related” federal agencies, to enable their cooperation and collaboration with agencies that are accountable for health care concerns, and to ensure that their policies are aligned to address diabetes and its complications in the United States.

## Data availability statement

The original contributions presented in the study are included in the article. Further inquiries can be directed to the corresponding author.

## Author contributions

Both authors contributed to the study conception and design. The first draft of the manuscript was written by WHH. Both authors edited, read, and approved the final manuscript.
